# Prevalence and factors associated with traditional herbal medicine use among patients on highly active antiretroviral therapy in Uganda

**DOI:** 10.1186/1471-2458-11-855

**Published:** 2011-11-10

**Authors:** Betty Namuddu, Joan N Kalyango, Charles Karamagi, Peter Mudiope, Samwel Sumba, Henry Kalende, Eric Wobudeya, Brian K Kigozi, Paul Waako

**Affiliations:** 1 Clinical Epidemiological Unit, Department of Medicine, College of Health Sciences, Makerere University, P. O. Box 7072, Kampala, Uganda; 2 Department of Pharmacy, College of Health Sciences, Makerere University, P. O. Box 7072, Kampala, Uganda; 3 Uganda Virus Research Institute, P.O. Box 49, Entebbe, Uganda; 4 Department of Pharmacology and Therapeutics, College of Health Sciences, Makerere University, P.O. Box 7072, Kampala, Uganda

## Abstract

**Background:**

In Africa, herbal medicines are often used as primary treatment for Human immunodeficiency virus (HIV) related problems. Concurrent use of traditional herbal medicines (THM) with antiretroviral drugs (ARVs) is widespread among HIV infected patients. However, the extent of THM use is not known in most settings in Sub-Saharan Africa. This study aimed at determining the prevalence and factors associated with THM use among HIV infected patients on highly active antiretroviral therapy (HAART) attending The AIDS Support Organization (TASO) in Uganda. TASO is a non-governmental organization devoted to offering HIV/AIDS care and treatment services in the population.

**Methods:**

This was a cross-sectional study carried out in two TASO treatment centres in Uganda among 401 randomly selected eligible participants. We included participants who were 18 years and above, were enrolled on HAART, and consented to participate in the study. Data was collected using an interviewer-administered semi-structured questionnaire. THM use referred to someone who had ever used or was currently using herbal medicine while on highly active antiretroviral therapy (HAART) by the time of the study. Data was captured in Epi-data version 3.1 and exported to STATA version 9.0 for analysis.

**Results:**

The prevalence of THM use was 33.7%. Patients on HAART for < 4 years were more likely to use THM (OR = 5.98, 95% CI 1.13 - 31.73) as well as those who experienced HAART side effects (OR = 3.66, 95% CI: 1.15 - 11.68). Older patients (≥39 years) were less likely to use THM (OR = 0.26 95% CI: 0.08 - 0.83). Participants with HAART adherence levels > 95% were less likely to use THM (OR = 0.09, 95% CI 0.01 - 0.65).

**Conclusion:**

The prevalence of THM use among participants on HAART was high. This raises clinical and pharmacological concerns that need attention by the health care service providers.

## Background

Worldwide, more than 40 million people are infected with Human immunodeficiency virus (HIV) [1] and more than 20 million deaths have been attributed to Acquired immunodeficiency syndrome (AIDS) since the onset of the HIV pandemic [2]. Globally there were over 3 million people in low middle income countries and 2 million in the Sub-Saharan Africa on HAART by 2002-2007[2]. In Uganda, there are over 1 million people with HIV and of these more than 312,000 need HAART, but only about 212,200 have access to the drugs[3].

The introduction of highly active antiretroviral therapy (HAART) has led to reduction in AIDS-related morbidity and mortality[4]. However the management of HAART side effects has remained a challenge in many resource limited settings[5], hence the increased use of complementary and alternative medicine (CAM).

CAM can be defined as any treatment used in conjunction (complementary) or in place of (alternative) standard medical treatment [6]. In Canada, complementary and alternative medicine (CAM) use, was most common in out-patients that reported HAART-related side effects (especially neuropathy)[7]. The World Health Organization (WHO) estimates that 4 billion people, which is 80% of the world's population, presently use herbal medicines for some aspect of primary health care in conjunction with conventional medicines [6]. In Africa, the majority of HIV patients rely on traditional herbal medicine (THM) for management of side effects and other primary health care needs[8,9]. Some studies have reported large numbers of traditional health practices (THPs) in the treatment of HIV/AIDS in developing countries involved in the treatment of HIV/AIDS [3]. In addition, some African nations such as South Africa, the Ministry of Health is currently promoting the use of traditional medicines for the treatment of HIV and associated symptoms [10] In the case of South Africa, the Ministry of Health is actively promoting the use of traditional medicines concurrently with antiretroviral treatments [10].

Concurrent use of THM and antiretroviral drugs may lead to drug interactions that may interfere with the effectiveness of HAART [11]. Previous studies have found that HIV-infected patients who distrust their health care providers are more likely to use CAM as a substitute for conventional HIV therapy[12]. In addition, HIV-infected patients with a greater desire for medical information and a negative attitude toward the effectiveness of antiretrovirals are more likely to use CAM with potential for adverse effects [12]. However, the extent and factors associated with herbal medicine use among HIV/AIDs patients on HAART is not well documented in Uganda. Consequently there are no clear guidelines on herbal medicine use among HAART patients in Uganda. This study was therefore conducted to determine the prevalence and factors associated with THM use among HIV infected patients on HAART attending The AIDS Support Organization (TASo) in Uganda.

### Study site description

TASO is a leading non-governmental HIV/AIDS organization with over 18,000 clients initiated on HAART by the end of 2007. It has 11 treatment centres country wide that provide free counseling and medical care to all people who seek their services. Each centre serves a population from a radius of about 75 kms.

## Methods

### Study design

A cross-sectional study was conducted among 401 patients on HAART at two TASO treatment centres in Jinja and Entebbe from January to March 2008.

### Subject Selection

The two sites were conveniently selected. The study participants from the centres were selected by simple random sampling method using MS EXCEL ("RAND function") computer generated random numbers. The patients' registration numbers from the HAART refill database for each centre were used to generate the random numbers. All numbers similar to those assigned to the patients' registration numbers were chosen until the required sample size was achieved. We selected 233 participants from Jinja and 168 from Entebbe. The sampled numbers were based on the clientele load at each site.

Patients were eligible for participation in our study if they were on HAART, were aged 18years and above, and consented to participate in the study. Seven patients refused to consent and 13 patients that had consented refused to respond to most of the study questions. The latter were replaced

### Data collection

Participant data were collected using a pilot-tested interviewer-administered semi-structured questionnaire developed specifically for the research. The data was collected by six trained research assistants (3 in each TASO centre) under direct supervision of the principal investigator. The outcome measure was traditional herbal medicine use. This referred to someone who had ever used or was currently using herbs that contain active ingredients of; parts of plants, or plant material or a combination of both while on HAART. Participants were asked when and why they started using THM, what herb was taken, how often they used it and whether they had disclosed this use to the clinicians.

Data was also collected on; demographic characteristics of the participants including age, sex, religion, highest level of education attained, occupation, marital status and religion; drug related factors including adherence to HAART, duration on HAART, and side effects; individual factors including familiarity with herbs, sexual activity, risky sexual behaviors, beliefs in the usefulness of THM, disclosure of HIV status, knowledge about HAART, and perceived health status while on HAART; clinical characteristics including CD4 cell counts, weight gain and having opportunistic infections; and health service related factors including type of information got from the counselors, method of counseling and patient doctor relationship.

Respondents were asked when and why they started using THM, what herb was taken and how often among others. We also tried to find out if THM use was disclosed to the clinicians. Medical and counseling records were crosschecked to verify the data got from the participants on the clinical, drug related and some of the individual factors like sexual behaviors

### Ethical issues

The study was approved by the Makerere University College of Health Sciences Medicine Research and Ethics Committee. The study included only patients that had given informed consent before data collection.

We ensured confidentiality of the participants' information using numbers instead of the participants' names as well as using persons that were not employed at these centers to collect the data.

### Statistical issues

Sample size calculation was based on the assumption that 19% [13] of HAART patients in Uganda with HAART related side effects use traditional herbal medicine, a significance level, of 0.05 and a power of 0.80. We computed sample size using Kish Leslie (1965) formula and Epi-Info STATCALC. In order to have adequate power and two-sided testing for associations, a minimum sample size of 400 was required.

The data was captured using EPI-data version 3.1(The EpiData Association, Odense, Denmark) and exported to STATA 9.0 (Stata, College Station, TX, USA) for statistical analysis. Descriptive statistics were used to summarize baseline characteristics and to determine the prevalence of THM use. Bivariate analysis was used to determine the factors associated with THM appropriately using either chi-square tests or Fisher's exact tests. A p-value less than 0.05, was considered significant. To determine factors that are independently associated with THM use, variables with P < 0.2 at bivariate analysis was entered into the logistic regression model for further analysis. We assessed for interaction using the chunk test to compare negative two log likelihoods of full and reduced models. We considered confounding present if there was a difference of at least 10% between crude and adjusted odds ratios. On the other hand, over 63% of the adherence data from the participants' individual files was missing and this could have affected our findings.

## Results

### Descriptive results

We enrolled 401 participants enrolled on HAART, 280 (69.8%) were women and the median age was 39 years (IQR 18.78) (Table 1). The majority of the respondents had attained primary level education, (n = 204, 50.9%). About thirty percent were married and almost half were self-employed (n = 195, 48.6%).

**Table 1:** Characteristics of patients on HAART at TASO Uganda, during 2008

Characteristic	Frequency	Percentage
**Women**	280	69.8
**Men**	121	30.2
**Median age (IQR)**	11	2.74
**Education level**		
**None**	54	13.5
**Primary**	204	50.9
**Secondary**	130	32.4
**Tertiary**	13	3.24
**Religion**		
**Catholic**	109	27.2
**Anglican**	132	32.9
**Muslim**	73	18.2
**Pentecost**	77	19.2
**Advents**	8	2.0
**Other**	2	0.5
**Marital status**		
**Single**	73	18.2
**Married**	138	34.4
**Divorced/Separated**	72	18
**Widowed**	118	29.4
**Occupation**		
**Self employed**	195	48.6
**Salary earners**	68	17.0
**Peasant farmers**	55	13.7
**Housewife**	23	5.7
**None**	59	14.7
**Student**	1	0.2

### Prevalence of THM use

The prevalence of THM use was 33.7%, (95% CI: 33.38-34.02), (Table 2). THM use was similar among patients at the two centers but was higher among women compared to men (36.4% versus 27.3% respectively).

**Table 2:** The prevalence of traditional herbal medicine use among patients on HAART at TASO Uganda, during 2008

Variable	Herbal users	Prevalence of THM use	95% CI
**Overall herbal use**	135/401	33.7	29.1 - 38.4
**Sex**			
Men	33/121	27.3	19.9 - 35.7
Women	102/280	36.4	30.9 - 42.1
**Age (categorized at median age)**			
≤ 38 years	56/211	26.5	25.8 - 27.2
≥ 39 years	79/190	41.6	41.0 - 42.2
**Religion**			
Christian	120/326	36.6	31.7 - 42.1
Muslim	15/73	20.5	12.4 - 30.9
**Marital Status**			
Single	101/263	38.4	32.6 - 44.3
Married	34/138	24.6	17.9 - 32.3
**Highest education level attained**			
Primary	69/204	33.8	27.5 - 40.5
Secondary	45/130	34.6	26.8 - 43.1
Tertiary	4/13	30.8	10.6 - 58.7
None	17/54	31.5	20.1 - 44.7
**Occupation**			
Employed	83/263	31.6	21.6 - 37.3
Not employed	52/138	37.7	29.8 - 45.9
**Location**			
Urban	74/206	35.9	25.9 - 42.6
Rural	61/195	31.3	25.0 - 38.0
**Site**			
Entebbe	57/168	33.9	27.0 - 41.3
Jinja	78/233	33.5	27.6 - 39.7

Reasons most stated for THM use were to reduce constant fever (67.4%) and treat cough (65.2%), (Table 3). Most of the herbs were obtained from registered herbalist (69.6%) and very few (5.2%) were obtained from pharmacies. Of the 135 patients on HAART who reported using THM, 130 (96.3%) perceived better health where the majority 63 (46.7%) got the herbs from the registered herbalists, (Table 4). The herbs used ranged from locally mixed stems, wood barks, leaves, food supplements and personal/individual morning urine (11.1%).

**Table 3:** Reasons and dosage for herbal medicine use among patients on HAART at TASO Uganda, during 2008

Variable	Frequency (N = 135)	Percent
***Reasons for herbal use		
Constant Fever	91	67.4
Too much Cough	88	65.2
General pain of HIV	71	52.6
Itchy skin	58	43.0
Others	58	43.0
To gain Energy	52	38.5
Was Anemic	39	28.9
Always fall sick	39	28.9
Low immunity	19	14.1
Loss/gain Appetite	18	13.3
Diarrhea	16	11.9
Too much sweat	11	8.1
**Herbal dosage per day**		
Half to one cup/glass	128	94.8
One to two spoons	60	44.4
No measurements	17	12.6
**Where herbs were got from**		
Herbalist	54	40
Garden	63	46.7
Friends/relatives	14	10.4
Pharmacy	4	7
**Experienced improvement with use of herbs		
Yes	130	96.3
Cannot tell	5	3.7
**Decision of herbal use**		
Friends	71	52.6
Parents/Relatives	38	28.1
Own decision/No one	26	19.3
**Patient's belief/perception of usefulness of ARVS**		
ARVs not yet helpful	35	25.9
ARVs helpful	100	74.1

** *including all those who had ever used herbs before and after initiation on HAART*

***Others include body swells (1.17%) vaginal wounds (1.01%), syphilis (1.17%), TB (0.67%), painful legs (1.17%), herpes (1%) and others

**Table 4:** Type of herbal medicine used to treat symptoms of HAART among patients at TASO Uganda, during 2008

Type of herb taken	Biological names	N = 135 (%)	Symptoms treated
Mpafu/jjlikiti/	*Canariu m schwcimf urthii*		Fever, Cough, Weakness
	*Erythrina abyssinica*		
Kasaana/			
Mwooloola/	*Entanda abyssinica*	115(85.2)	
Ovakedo	*Bersea Americana*		
	*P s i d i um guajava*		
Amapeera	*Mangifera in dica*		
Miyembe (leaves)			
Other Herbs	Just mentioned as "others"	86(63.7)	Joint pains, Fever, cough
Kadomola mixture	Can be mixture of food juice, or bark trees and s t e m s	84(62.2)	Fever, Cough, oral thrash, stomach wounds, HIV pain, appetite, Diarrhea, body heat
Plant roots and stems boiled		78(57.8)	Fever, cough, back ace
Kigagi	Aloe Vera	76(56.3)	Fever
Don't know name of herb		62(45.9)	Fever, body heat, energy
Bazukuza Bafu	*Hibiscus*	61(45.2)	Anemia
Kamunye Herb	*Hoslundia opposite*	41(30.4)	Fever, stomach wounds
Molinga Herb	*Oleifera*	35(25.9)	Fever, weakness, appetite
Muzukizi Herb	*Dicliptere laxata*	33(24.4)	
Kamwanyi Herb		31(23.0)	Fever
Mululuza Herb	*Vernonia a mygdalina*	29(21.5)	Fever
Boombo Herb	*Memodica f eo tida*	26(19.3)	Cough
Naloongo Herb	*Justicia betonica*	21(15.6)	Fever
Kikakala Herb		21(15.6)	
Nim tree Herb		20(14.8)	
Kanzironziro Herb	*P sorospermum febrifugum*	20(14.8)	Body weakness
Olubirizi Herb	*Vernonia a mygdalina*	18(13.3)	
Musita Herb	*Albizia cariaria*	17(12.6)	
Nakakasero Herb		16(11.9)	Fever
Own Urine (morning personal urine)	-	15(11.1)	HIV generally
Ngetwa Herb	Consists of 54 herbs (mainly from Tanzania)	15(11.1)	Cough, fever, appetite, stomach pain, headache
Olusazisazi Herb	*Cassia occidentalis*	12(8.9)	
Kambula Herb	*Cardiospermu m granflor um*	9(6.7) 7(5.2)	
Herbal Soap and jelly	-		Skin rash
Pressure herbs	-	4(3.0)	High blood pressure,

Most of the herbs were boiled and orally administered

### Bivariate analysis

Single individuals were more likely to use THM compared to the married individuals (P = 0.006), (Table 5). The participants who were ≥ 39 years were less likely to use THM than those who were ≤ 38 years, (P = 0.002). Participants who reported side effects from HAART were two times more likely to use THM compared to those who did not, (Table 6). Individuals who had been on HAART for < 4 years were more likely to use herbs than those who were on HAART for ≥ 4 years, (P = 0.048). Patients who could not tell if herbs actually improved health were more than three times more likely to use THM compared to those who could tell it improves on the health of an individual.

**Table 5:** Bivariate analysis of socio-demographic factors and herbal medicine use among patients on HAART at TASO Uganda, during 2008

Variable	Used herbal N = 135(%)	Not used herbal N = 266 (%)	Unadjusted OR (95%CI)	P- value
**Sex**				
Male	33 (24.4)	88 (33.1)	1.00	**0.076**
Female	102 (75.6)	178 (66.9)	0.65 (0.41 - 1.05)	
**Age (categorized at median age)**				
≤ 38 years	56 (41.5)	155 (58.3)	1.00	**0.002**
≥ 39 years	79 (58.5)	111 (41.7)	0.51 (0.33 - 0.77)	
*Religion				
Catholic	42 (31.11)	67 (25.19)	1.59 (0.097 - 26.1)	0.74
Protestant	49 (36.30)	83 (31.20)	1.69 (0.01 - 27.6)	0.71
Muslim	15 (11.11)	58 (21.80)	3.86 (0.2 - 65.4)	0.34
Pentecost	24 (17.78)	53 (19.92)	2.2 (0.13 - 36.8)	0.58
Other	5 (3.70)	5 (1.88)	1.00	
**Marital Status**				
Married	34 (25.2)	104 (39.1)	1.00	**0.006**
Not married	101 (74.8)	162 (60.9)	1.91 (1.20 - 3.02)	
**Highest education level attained**				
Primary	69 (51.11)	135 (50.75)	1.00	
Secondary	45 (33.33)	85 (31.95)	0.97 (0.61 - 1.54)	0.882
Tertiary	4 (2.96)	9 (3.38)	1.15 (0.34 - 3.88)	0.822
None	17 (12.59)	37 (13.91)	1.11 (0.58 - 2.12)	0.746
**Occupation**				
Self employed	62 (45.93)	133 (50.00)	1.00	
Salaried employed	21 (15.56)	47 (17.67)	1.04 (0.57 - 1.90)	0.889
None	30 (22.22)	53 (19.92)	0.82 (0.48 - 1.41)	0.481
Peasant	22 (16.30)	33 (12.41)	0.70 (0.38 - 1.30)	0.256

*overall p value 0.11

**Table 6:** Bivariate analysis of drug related and individual factors associated with THM use among patients on HAART at TASO, during 2008

Variable	Used herbal N = 135(%)	Not used herbal N = 266 (%)	Unadjusted Odds ratio (95%CI)	P- value
**Duration on HAART**				
≥ 4 years	25 (18.5)	30 (11.3)	1.00	**0.064**
< 4 years	110 (81.5)	236 (88.7)	1.79 (1.00 - 3.18)	
**Ever experienced side effects**				
No	50 (37.0)	146 (54.5)	1.00	**0.001**
Yes	85 (63.0)	121 (45.5)	2.04(1.33 - 3.12)	
Adherence level in the last 3 months*				
75% - 95%	3 (6.1)	8 (9.4)	1.00	0.74
> 95%	46 (93.9)	77 (90.6)	0.6 (0.1 - 2.4)	
**Duration on herbs while on HAART**				
< 6 months	30 (22.22)	35 (43.8)	1.00	
6 - 12 months	35 (25.93)	16 (20.0)	0.39 (0.18 - 0.86)	**0.016**
More than a year	70 (51.85)	29 (36.3)	0.35 (0.18 - 0.70)	**0.002**
**Ever changed regimen**				
Yes	39 (28.89)	45 (16.92)	1.00	
No	96 (71.11)	221 (83.08)	2.00 (1.21 - 3.28)	**0.006**
**Herbs improved health**				
Yes	130(96.2)	64()	1.00	
Cannot tell	5(3.8)	16(6.02)	2.64 (1.71 - 4.08)	**P < 0.001**
**Familiar with herbs**				
Yes	135 (100)	218 (82.0)	1.00	**P < 0.001**
No	0	48 (18.0)	0.62 (0.577 - 0.67)	
**Outcome expectation of Herbs**				
Yes	135 (100)	62 (77.5)	1.00	**P < 0.001**
No	0	18 (22.5)	0.32 (0.25 - 0.39)	
Sexually active patients*				
Yes	100 (74.60)	120 (62.50)	1.00	
No	34 (25.40)	72 (37.50)	0.57 (0.35 - 0.92)	**0.023**
**Disclosure of HIV to others Status**				
Yes	119 (88.1)	207 (77.8)	1.00	
No	16 (11.9)	59 (22.2)	2.12 (1.16 - 3.85)	**0.012**

*Missing values

### Multivariate analysis

The risk factors that were independently associated with a decrease in THM use included; Young patients (≤38 years), HAART adherence levels ≤ 95%, patients on HAART for < 4 years, participants who experienced HAART side effects, not sexually active and those who could not tell that THM improves on the health of an individual and statistical significance was achieved (Table 7).

**Table 7:** Multivariate model for factors associated with herbal medicine use among patients on HAART at TASO, during 2008

Variable	Adjusted OR	95% CI	P-value
**Age categorized (median)**			
≤ 38 years	1.00		
≥ 39 years	0.26	0.08 - 0.83	0.023
**ART Adherence level**			
75% - 95%	1.00		
>95%	0.09	0.01 - 0.65	0.017
**Herbs improved health**			
Yes	1.00		
Cannot tell	2.40	1.62 - 3.55	0.001
**Duration on HAART**			
≥ 4 years	1.00		
< 4 years	5.98	1.13 - 31.73	0.036
**Ever experienced side effects**			
No	1.00		
Yes	3.66	1.15 -11.68	0.028
Sexually active participant*			
Yes	1.00		
No	0.26	0.08 - 0.90	0.033

## Discussion

The prevalence of traditional herbal medicine use among patients on HAART in TASO Uganda was high with one in every three patients using herbs alongside HAART. However, this prevalence of THM use may be an underestimate of the true prevalence because we used self-reports to assess herbal medicine use. Some patients may not have reported herbal medicine use alongside HAART since it is usually discouraged by the health care providers. Our study was done in a setting where patients are sensitized on the dangers of combining conventional drugs like HAART with THM and patients are followed up to monitor their progress. The training of traditional herbalists in good practices of herbal dispensing may promoted THM use hence high prevalence. The training was conducted by the WHO in association with the Tradition and Modern Health Practitioners together with HIV/AIDS and other Diseases (THETA), community based organization in Uganda.

Most of the literature reports CAM among HIV patients with THM being just a part of CAM [14]. This makes it difficult for us to make good comparison. Nevertheless, other studies show a higher prevalence of CAM than the THM we found in our study. In British Colombia the prevalence of herbal medicine use among patients on was 19%[15]. The prevalence of THM use in our study is lower than that found in the USA where 67% of HIV patients were taking herbs, of which 20% were taking Chinese herbs, in one study [14] and less than 8% in Eastern Massachusetts and Rhode Island[16]. The later however, considered both patients on HAART and those not on HAART. The latter are likely to use THM more. In Canada, 30% of the HIV out patients reported use of herbs, [17]. THM like CAM use was high among the HIV-positive population and was primarily used to improve general health in the USA [14,16,18]. The general lower prevalence of herbal medicine use among HIV patients in the developed world is reflected by the low prevalence (14%) of herbal/supplemental use in the population [14,16,18].

In our study, the prevalence of THM use among the women was higher than that in men, but this was not statistically significant. A similar trend has been observed in large surveys in the USA[14,16,18]. Age was associated with THM use in our study, and was statistically significant. This is in contrast to findings from the study in British Columbia[15] where there was no association between age and THM use.

It is not surprising that, individuals who were on HAART for a relatively short period of time also use THM more than those who on HAART for more than four years in our study. This is in contrast to a study by Agnoletto et al where patients with longer duration on HAART are more likely to use CAM[7]. It is possible that these patients had not yet been well sensitized about not using herbs alongside HAART or they had been using THM before they were enrolled on HAART.

Our study showed HAART side effects like anemia and HIV related symptoms (fever, cough, joint pain, oral thrash, etc.), were the main reasons for THM use. This is possibly due to the information provided by traditional herbalists that THM are natural foods without any toxic element that give good relief if eaten in good quantities. There is Increasing proof of the interactions of herbs/supplement and HAART with potential for serious adverse effects[11,12]. Some herbs (e.g., germander, comfrey, pennyroyal) have well-documented toxic effects[19]. Our study could not clearly establish the adverse effects of THM since we mainly focused on THM use. However a study by Hsiao et al[12] in the United States focused on potential adverse effects found one quarter of the CAM to have adverse effects. THM and HAART may share similar adverse effects. Patients using both therapies may wrongly attribute their adverse effects to the antiretroviral. Interactions of this type may result in increased toxicity and decreased efficacy of both THM and HAART[20]. We found out that some clinicians also advise and prescribe patients to use the anemia herb. Clinicians who are not aware of their patients' use of THM may change antiretroviral regimens unnecessarily.

Participants' self-report on improved health status with THM, before and after enrolling on HAART could possibly be true. On the other hand, it could be due to HAART as well as care and support provided by the counselors and clinicians at the treatment centres. This was referred to as positive outcome due to herbal use, but was not clinically assessed by the study. "It is not unreasonable to suggest that some herbal products may have therapeutic benefits", Edward Mills reported[21]. A randomized clinical trial in China reported promising effects from three herbal medicines and combined treatment of herbal medicine and ARV agents showed increased antiviral benefits compared to ARVs alone[5]. In addition, almost no pharmacokinetic interaction studies so far have been conducted with HIV-infected patients[11]. Pharmacological investigations would be prudent to identify the potential risks, benefits, and interaction or noninteractions associated with HAART and local THM use in Uganda.

Adherence to antiretroviral medication in the treatment of HIV is critical, both to maximize efficacy and to minimize the emergence of drug resistance[22]. Patients whose HAART adherence level was below 95% were more likely to use THM. This is similar to what Kiguba et at[13] found out in Uganda, although his outcome was not THM as in our study. In Zambia, half of the pregnant women with low adherence to Nevirapine were using THM [23]. In KwaZulu-Natal, South-Africa, results indicated that the use of herbal therapies for HIV declined significantly from 36.6% prior to HAART initiation to 7.9% after being on ARVs for 6 months[24]. However, the study designs and adherence measureme nt were different, hence not comparable.

## Limitations of the study

Study participants were well informed about HAART making them a unique population. This would be different if study was done on a different population for example in general hospitals that provide HAART in Uganda, where emphasizes on the dangers of mixing conventional drugs with THM is very minimal. On the other hand THM use was self-reported. It is plausible that some respondents were hesitant to report actual THM use, and possibly fearing to be reprimanded or denied further HAART care. To some extent, this could have biased or resulted to an underestimation of our results, because, if one reported use, then he actually did combine the THM and HAART. It is plausible that we would have got more accurate results, if the interviews were not carried out at the treatment Centre. Adherence was not fully assessed due to missing and/or incomplete data. Most patients reported weight gain after using herbs for some time. We could not verify this self-report for our study design could not enable us measure causality.

## Conclusions

There is a wide spread use of traditional herbal medicines concurrently with antiretroviral drugs. Potential beneficial effects need to be confirmed in large, rigorous designed trials or large survey researches. Although guidelines of herbal use is still lacking in the country, it is important that herbal medicine use be monitored among HAART patients in case of any drug interactions or resistance.

## Competing interests

The authors declare that they have no competing interests.

## Authors' contributions

BN: Study concept and design, queried and extracted data from TASO database, data assembly, analysis and interpretation of the findings and wrote-up the manuscript. JNK: assisted in the study concept and design, interpretation of findings and critical revision of the final approval of the manuscript. CK, PM, SS, HK: assisted in data analysis, interpretation of study findings and critical revision of the final manuscript. EW, BKK: assisted in the interpretation of findings, critical revision of the final approval of manuscript. WP: assisted in the study concept and design, interpretation of findings and critical revision of the manuscript.

All authors read through the final manuscript and accepted the changes made.

## Pre-publication history

The pre-publication history for this paper can be accessed here:

http://www.biomedcentral.com/1471-2458/11/855/prepub
